# Ectopic Tooth in the Maxillary Sinus: A Rare Case

**DOI:** 10.7759/cureus.93115

**Published:** 2025-09-24

**Authors:** Yusuf Ç Kumbul, Berkay Kuscu, Alperen Uzumcu, Furkan Ozturk

**Affiliations:** 1 Otolaryngology - Head and Neck Surgery, Süleyman Demirel University School of Medicine, Isparta, TUR

**Keywords:** endoscopic sinus surgery (ess), maxillary sinus, odontogenesis, tooth, tooth abnormalities

## Abstract

The presence of an ectopic tooth in the maxillary sinus is a rare condition. The term ectopic tooth refers to a clinical condition marked by the presence of a tooth in an atypical location, away from its normal anatomical site. In the present case, the surgical removal of an ectopic tooth in the left maxillary sinus is described in a 24-year-old woman. This article discusses possible etiological factors, symptomatology, the role of imaging methods, and treatment approaches in the context of the literature.

## Introduction

The ectoderm of the oral cavity, which produces cells that make enamel, and the neural crest ectomesenchyme, which produces the tooth components other than enamel, interact to create the intricate process of tooth development. At any stage of this process, ectopic tooth (ET) development and eruption can be caused by abnormal interactions [1,2]. The term ET refers to a clinical condition marked by the presence of a tooth in an atypical place, away from its normal anatomical site [3]. In addition, ectopic eruption is a condition in which, because of deficient growth in the jaw or a segment of the jaw, a primary tooth assumes a path of eruption that leads to its premature loss and produces a consequent malposition of the permanent tooth [4]. These atypical anatomical regions may include the maxillary sinus, mandible, palate, and nasal cavity [3,5].

ET formation is not a common health problem for a certain age group. It is a condition that affects a wide age range from children to adults. The main reason for this condition has not yet been clearly explained. Although it is quite difficult to notice the presence of ET in the maxillary sinus, most patients present to the hospital with complaints such as nasal congestion, pain radiating to the face, headache, snoring, postnasal drip, and runny nose [6]. In addition, patients may receive an incidental diagnosis of ET as a result of radiological imaging without any complaints, as in the case we present.

In this article, we aimed to present the diagnosis and treatment process of a 24-year-old female patient in whom an incidental ET was detected in the maxillary sinus, accompanied by a review of the literature. An informed consent form was obtained from the patient for this case report.

The present case report was presented as a poster presentation at the 2nd Project and Science Days held at Süleyman Demirel University Faculty of Medicine on May 7, 2025.

## Case presentation

**Figure 1:**
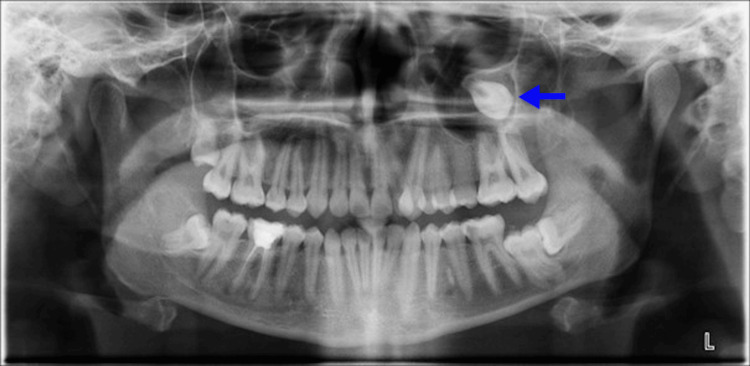
Ortopantomogram Blue arrow indicates the bone density lesion within the left maxillary sinüs in ortopantomogram.

A 24-year-old female patient presented to the orthodontic clinic with a complaint of dental crowding. During the examination, a bone density lesion was observed in the left maxillary sinus on the orthopantomogram, and she was referred to the otorhinolaryngology department (Figure 1).

**Figure 2:**
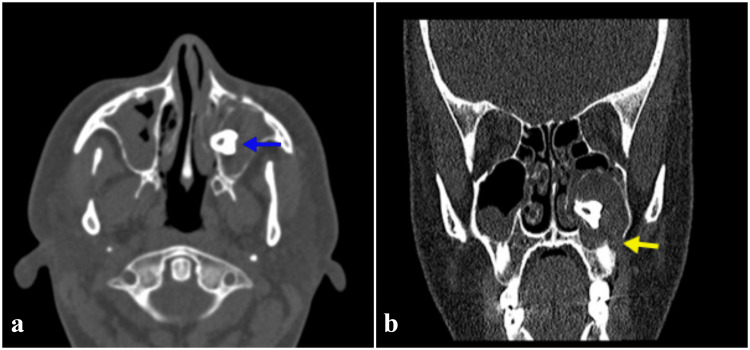
Paranasal Sinus Computed Tomography Images (a) Axial section; the blue arrow indicates the maxillary third molar tooth within the left maxillary sinus. (b) Coronal section; the yellow arrow indicates the defect in the left maxillary sinus inferolateral wall.

There were no features in the patient's complaint regarding otorhinolaryngologic diseases. On physical examination, the nasal septum was deviated to the left, the bilateral inferior turbinates were hypertrophied, and other ENT examinations were normal. The patient stated in her medical history that she had undergone surgery once due to angiomyolipoma, that she had asthma, and that she smoked one pack of cigarettes per day. In addition, the patient had a history of a tap battery falling from a height onto her forehead seven to eight years ago. As a result, there was a scar approximately 2 cm long just medial to her right eyebrow. The patient's family history was unremarkable. According to the patient's paranasal sinus computerized tomography, a bone-density lesion was detected in the left maxillary sinus, with a cystic wall surrounding this lesion and a bone defect in the inferolateral wall of the sinus (Figure 2). 

**Figure 3:**
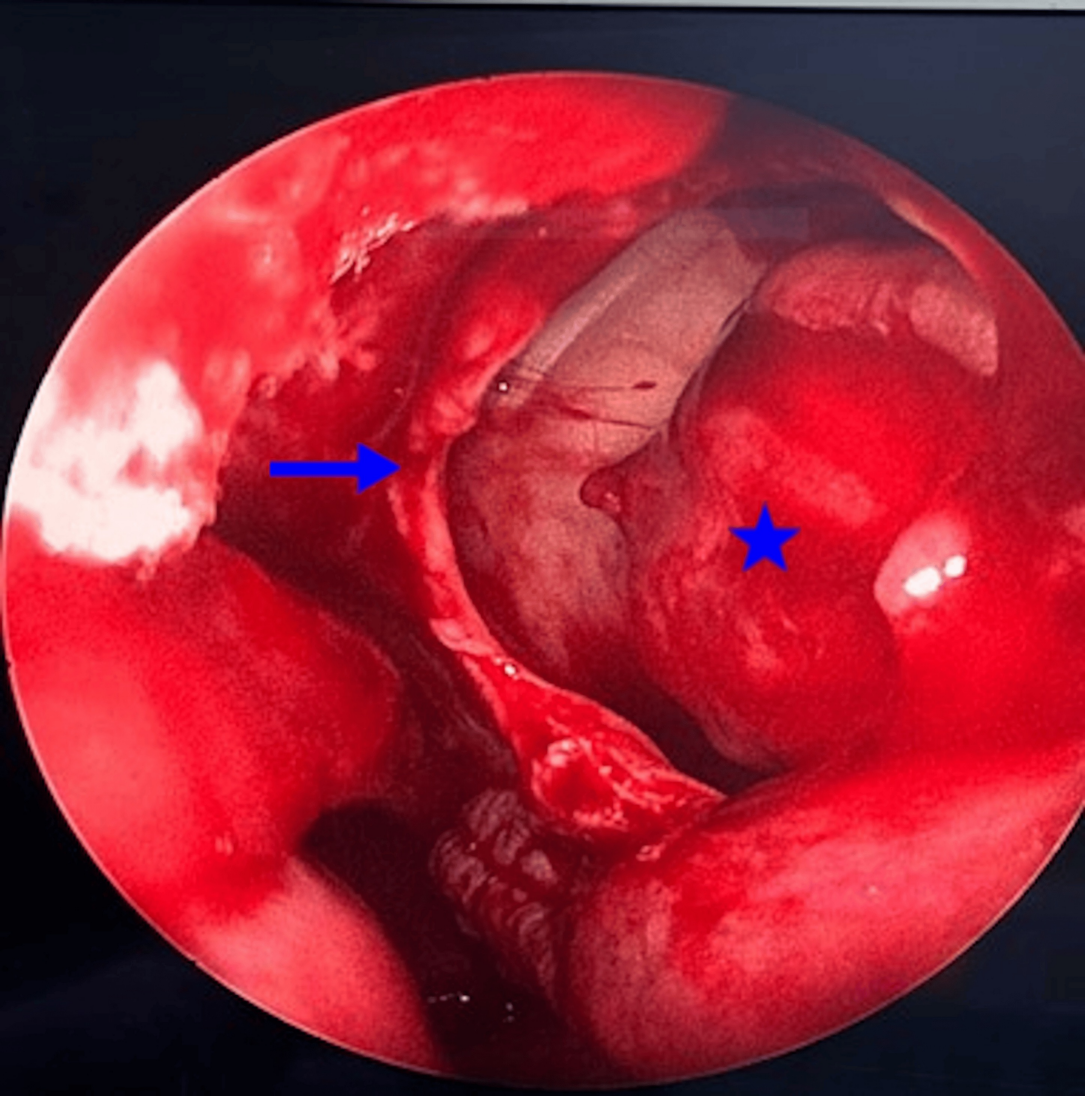
Intraoperative Image of the Lesion The blue arrow indicates the posterior wall of the left maxillary sinus ostium after uncinectomy and maxillary sinus ostium expansion. The blue star indicates the wall of the cystic formation within the sinus.

The patient received a preliminary diagnosis of an incidentally observed ET with the help of physical examination and imaging methods. It was planned that the patient would have the ET removed from the maxillary sinus with endoscopic sinus surgery after submucous resection for septum deviation. During the operation, first, submucous resection of the nasal septum was performed, and the nasal septum deviation was corrected. After uncinectomy and enlargement of the left maxillary sinus ostium were performed, wide exposure was provided into the sinus, and the lesion covered with a cyst wall was observed (Figure 3).

**Figure 4:**
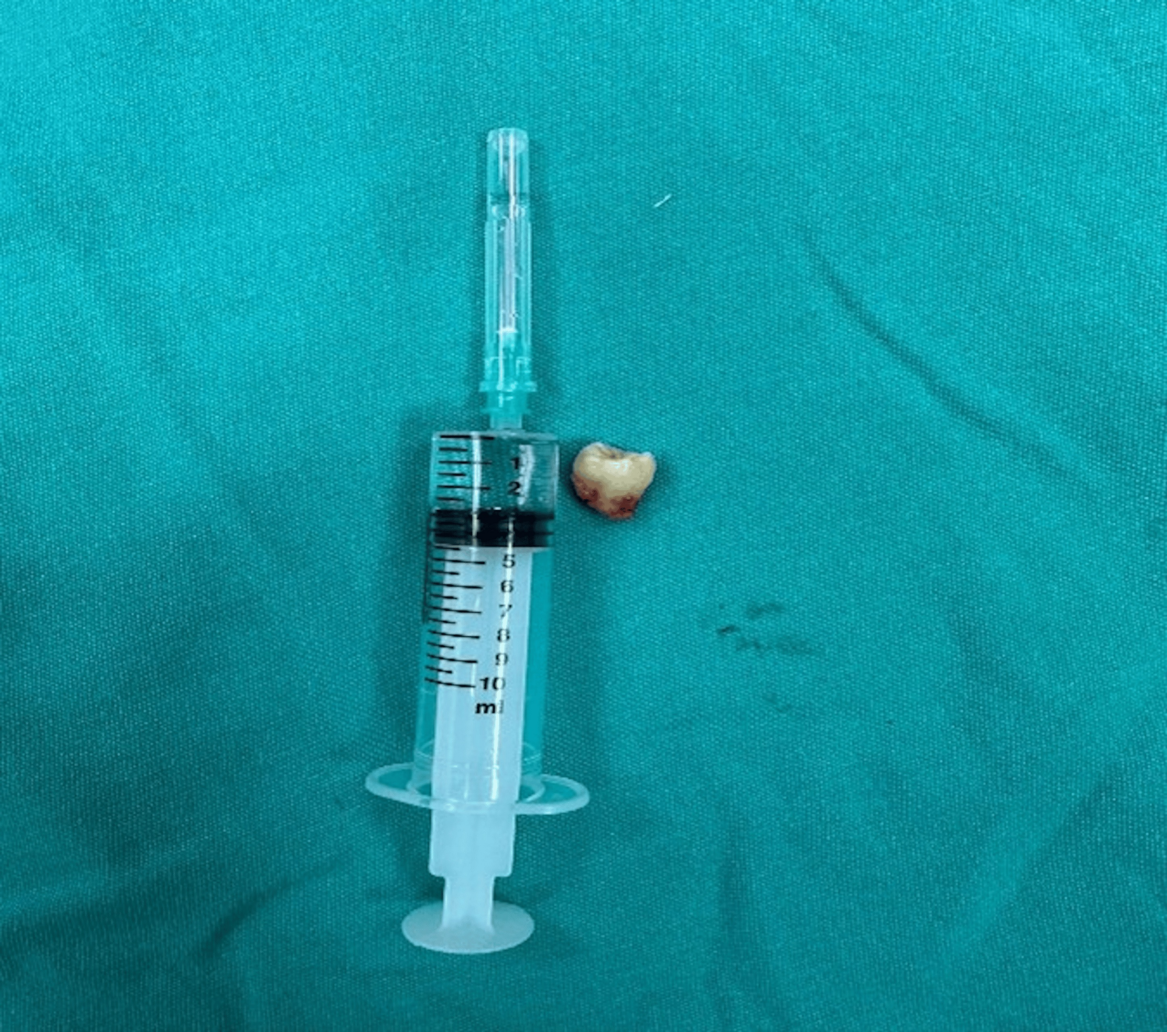
Tooth Excised From the Sinus Image of the maxillary third molar tooth removed from the cystic structure in the left maxillary sinus.

The lesion with the cyst wall and the ET structure (Figure 4) within the cyst were removed to form two separate pathology specimens. Then, the maxillary sinus walls were examined with the help of 0- and 30-degree endoscopes. The operation was terminated after the maxillary sinus walls were observed to be normal.

The patient, who did not develop any postoperative complications, was discharged on the first postoperative day with a prescription for cefdinir 600 mg (Ceftinex 600 mg, Bilim İlaç, İstanbul) 1×1 and paracetamol 500 mg (Parol 500 mg, Atabay İlaç, İstanbul) 3×1. The patient came for follow-up on the tenth postoperative day and had no complaints, and her endoscopic nasal examination was normal. The patient's pathology result was reported as a ciliated epithelial cyst and dental tissue. Thus, the patient's treatment process was completed.

## Discussion

The cause of ectopic eruption is still unknown; however, several possibilities have been proposed, including trauma, infection, pathological conditions, crowding, and developmental defects [7]. In a study of 14 cases with ET in the maxillary sinus, crowding was the most commonly implicated etiological factor, detected in 5 cases. The reason for this was given as the proximity of the ET in the maxillary sinus to the alveolar process in the cases presented [6]. In the case we present, there were suspected etiological factors such as crowding of teeth and a history of trauma to the maxillofacial region in the past. However, we could not clearly prove which of these factors caused the formation of ET.

Since the majority of instances are asymptomatic, ET in the maxillary sinus is typically found during routine clinical or radiological tests. Although patients have sinonasal complaints such as nasal congestion, snoring, postnasal drip, and pain that cannot be localized in the facial area, the causes of these complaints are diseases such as septum deviation, turbinate hypertrophy, and allergic rhinitis, and these patients are considered asymptomatic. However, if there is an osteomeatal complex obstruction associated with ET, chronic rhinosinusitis findings occur [6]. We believe that the most important point in the emergence of complaints related to ET in the maxillary sinus is obstruction caused by the ET in the osteomeatal complex.

There may be three types of ET in the maxillary sinus: permanent, deciduous, and supernumerary. Another clinical data point relates to which tooth is found as ET in the maxillary sinus. A study determined that with a rate of approximately 35%, the third molar tooth was the most common ET. In the same study, the second most common ET in the maxillary sinus was the first molar tooth. The ET in this case study was a third molar tooth, in accordance with the literature [6].

When the age and gender distribution of the cases is examined, the average age of diagnosis is 25.3 years, and female patients are more common [6]. In another study examining the ectopic placement of the third molar tooth in the mandible, 45 case reports were evaluated, and the average age of diagnosis was found to be 46.3 years. In the gender distribution of the same study, there was a clear predominance of females over males [8]. The difference in the location of the ET may have caused a difference in the age of diagnosis in these two studies. However, since these are rare cases, we believe that the age and gender predominance of diagnosis will be clearly demonstrated in larger case series or systematic reviews. Therefore, we believe that each case report will contribute to the literature.

Imaging methods are necessary to make a preliminary diagnosis of ET, to confirm the diagnosis, and to plan treatment. The most commonly used methods are Water’s radiography, orthopantomogram, and computerized tomography. When an ET is suspected based on imaging, the differential diagnosis should include the following diseases: rhinoliths/sinoliths, compound odontoma, complex odontoma, ameloblastic fibroodontoma, calcifying odontogenic cyst, calcifying epithelial odontogenic tumors, cementoossifying fibroma, infections with calcifications, and dermoid cysts. The major characteristic of ET is the ectopic location of the tooth outside the alveolar complex, and it may be associated with dentigerous cysts. Imaging indicates the presence of a tooth-like bony structure outside the alveolar complex [9]. We routinely recommend computed tomography for preoperative evaluation of ET, especially those to be removed from the maxillary sinus by endoscopic sinus surgery.

The definitive treatment for ET is surgical removal. Although the present case was asymptomatic, cyst formation around the ET and osteomeatal complex obstruction were our main surgical indications. The Caldwell-Luc procedure and the endoscopic maxillary sinus approach can be used for the removal of maxillary ET. The Caldwell-Luc surgical procedure is more invasive than endoscopic sinus surgery. It is a surgical method that is still used to gain control of the anterior and floor walls of the maxillary sinus [2]. In contrast, the angled endoscopes used in endoscopic methods provide sufficient exposure for the removal of the ET in the maxillary sinus, as in our case. In endoscopic approaches, where recovery is generally rapid, hospital discharge may be early. Apart from surgical removal, close radiological follow-up can be performed for asymptomatic isolated teeth [10] because, in these cases, recurrent or chronic rhinosinusitis, dentigerous cyst formation, tumors, nasal cavity obstruction, and orbital floor perforation may develop [11]. Radiological follow-up can also be performed to detect any recurrence after surgery.

## Conclusions

ET in the maxillary sinus is a rare condition. ET may remain asymptomatic, or sinonasal symptoms may be encountered. Dentists and otolaryngologists should be aware of this rare condition for differential diagnosis. Although the definitive treatment method is surgery, asymptomatic isolated teeth can also be followed radiologically.
